# Recent Advances of Fluorescent Aptasensors for the Detection of Antibiotics in Food

**DOI:** 10.3390/bios15040252

**Published:** 2025-04-16

**Authors:** Zheng Liu, Wenyi Yang, Huikai Lin, Mingdi Zhang, Chunyan Sun

**Affiliations:** Department of Food Quality and Safety, Jilin University, Changchun 130062, China

**Keywords:** fluorescent, aptasensors, dyes, nanomaterials, antibiotics, food

## Abstract

Antibiotics can accumulate in the body via ingestion, presenting serious health and safety risks to humans, and have garnered extensive international attention in recent years. Meanwhile, aptamers have been applied in the detection of antibiotics, mainly because of their good stability, high specificity, easy synthesis, and low cost. Among various kinds of aptasensors, fluorescent dye-based or nanomaterial-based fluorescent aptasensors serve as highly efficient tools for the rapid quantification of antibiotics owing to their remarkable sensitivity, specificity, and selectivity. In addition, some novel techniques such as aptamer tailoring, signal amplification, and artificial intelligence for aptasensors are also presented. This paper provides a detailed and comprehensive review of fluorescent aptasensors for antibiotic detection. Moreover, it pinpoints the challenges encountered during the development of the aforesaid fluorescent aptasensors and puts forward future research directions.

## 1. Introduction

**Figure 1:**
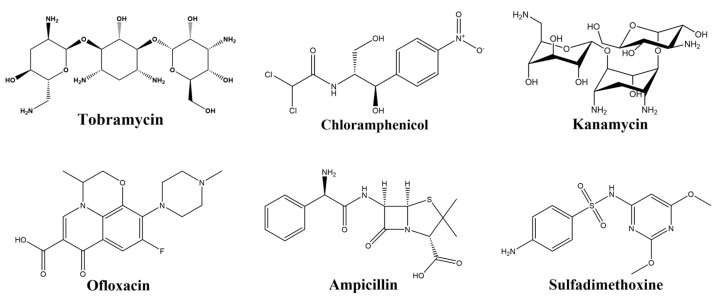
Chemical structures of different antibiotics.

Antibiotics are organic compounds produced by specific microorganisms during life that have bacteriostatic or bactericidal actions. They are divided into tetracyclines (TCs), aminoglycosides (AGSs), fluoroquinolones (FQs), β-lactams (BLCs), macrolide antibiotics, phenolic class (phenols), and sulfonamide (SA) antibiotics, which kill bacteria, stop their growth, and are also used to prevent or treat bacterial infections [1] (Figure 1). They also have applications in the medical industry, such as treating respiratory infections, orthopedics, surgery, neurological disorders, and even cancer [2]. However, long-term consumption of foods produced with overuse of antibiotics in agriculture can lead to side effects such as drug resistance, impaired liver function, and ototoxicity. Therefore, identifying a suitable technology for quick and accurate quantitative analysis of antibiotic levels, especially in foods and water environments, is of great significance to ensure food safety and human health.

In recent years, a large number of works have been performed to develop qualitative/quantitative methods for analyzing antibiotics. At present, antibiotic residue detection methods mainly include high-performance liquid chromatography (HPLC) [3], gas chromatography–mass spectrometry (GC-MS) [4], and liquid chromatography–tandem mass spectrometry (LC-MS/MS) [5]. These detection methods have excellent detection performance but often require huge equipment, high cost, and skilled personnel, which limits real-time detection and monitoring of on-site threats and other applications.

Aptamer technology has recently become a pioneer in the rapid detection of food contaminants. An aptamer is a single-stranded oligonucleotide (DNA or RNA) designed by an artificial process, called the systematic evolution of ligands by exponential enrichment (SELEX), with a high degree of specificity and affinity for its targets [6,7,8]. Unlike antibodies, aptamers can be chemically synthesized, so a variety of signal output elements are specially labeled and read by signal transducers, thus speeding up analysis time and reducing analysis costs [9]. In addition, they have a wide target range, good repeatability, high availability, easy production, and excellent stability [10] (Table 1). These excellent advantages promote the widespread use of aptamers as reliable recognition probes in biosensors. Based on different signal transduction modes, such as electrochemistry [11], fluorescence [12], colorimetry [13], etc., various aptasensors have emerged at the right moment. Compared to other aptasensors, fluorescent aptasensors have been widely used for the determination of biological interactions because of their benefits of flexible quantitative analysis, high sensitivity, easy application, and wide response range.

**Figure 2:**
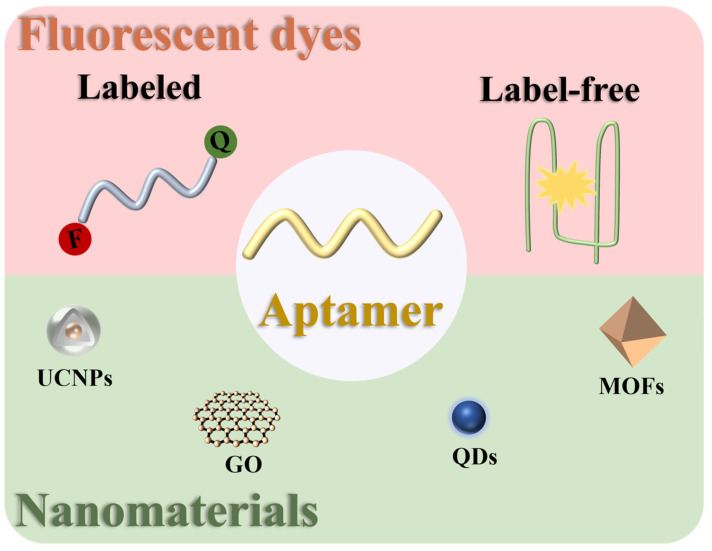
Schematic overview of fluorescent aptasensors for the detection of antibiotics in food.

In the past decade, the detection of antibiotics has been a research hotspot in the field of aptasensors. The average number of papers on this topic reached about 200 each year, of which fluorescent aptasensors accounted for about a quarter. So far, only a few reviews have presented recent work on aptamer-based detection of antibiotics [14,15]. This paper reviews the progress of research on the detection of antibiotics based on fluorescent aptasensors in the last few years. They can be mainly divided into two categories: fluorescent aptasensors based on fluorescent dyes and fluorescent aptasensors based on nanomaterials (Figure 2). In addition, we highlight the advantages and limitations of fluorescent aptasensors and evaluate their future challenges and development trends.

**Table 1:** Antibiotic-binding aptamers published in the literature.

Targets	Aptamer Sequences (5′-3′)	Reference
Tobramycin	GGGACTTGGTTTAGGTAATGAGTCCC	[12]
Chloramphenicol	ACTTCAGTGAGTTGTCCCACGGTCGGCGAGTCGGTGGTAG	[16]
Kanamycin	TGGGGGTTGAGGCTAAGCCGA	[17]
Tetracycline	CGTACGGAATTCGCTAGCCCCCCGGCAGGCCACGGCTTGGGTTGGTCCCACTGCGCGTGGATCC	[18]
Ofloxacin	ATACCAGCTTATTCAATTGCAGGGTATCTGAGGCTTGATCTACTAAATGTCGTGGGGCATTGCTATTGGCGTTGATACGTACAATCGTAATCAGTTAG	[19]
Sulfadimethoxine	GAGGGCAACGAGTGTTTATAGA	[20]
Sulfadimidine	TTAGCTTATGCGTTGGCCGGGATAAGGATCCAGCCGTTGTAGATTTGCGTTCTAACTCTC	[21]
Ampicillin	CTGAATTGGATCTCTCTTCTTGAGCGATCTCCACA	[22]

## 2. Fluorescent Dyes in Fluorescent Aptasensors for the Detection of Antibiotics

Fluorescent dyes are polyaromatic or heterocyclic hydrocarbons that are capable of emitting photons after being excited. The large Stokes shift of ideal fluorescent dyes for biomacromolecule detection is important since it helps to minimize the reabsorption of released photons and improves stability and biocompatibility. Fluorescent dyes have been widely employed to obtain readout signals in many detection and biological imaging applications because of their benefits of speed, sensitivity, low cost, easy recognition, and wide biocompatibility. According to whether the fluorescent dye is labeled on the aptamer, it can be divided into the labeled type and label-free type. An overview of the different fluorescent aptasensors based on fluorescent dyes for the detection of antibiotics in food can be found in Table 2.

### 2.1. Labeled Type

Before and after binding with the target, aptamers are known to have distinct conformations/structures. The conformational and/or structural modifications of aptamers can impact the fluorescence of a dye or the fluorescence resonance energy transfer (FRET) between two dyes by direct modification with fluorophores. The extent of the binding process can be reflected in the signal change, which can either rise (the “turn-off” type) or decrease (the “turn-on” mode), allowing for quantitative detection of the target concentration.

**Figure 3:**
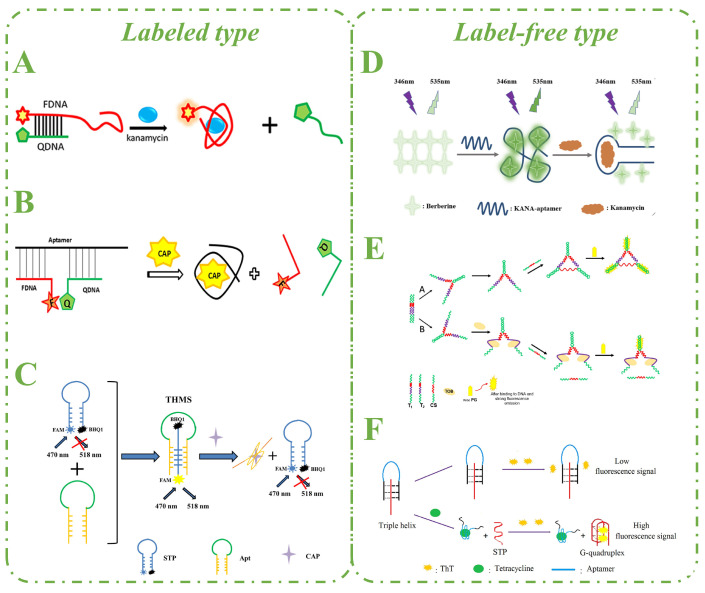
Labeled and label-free fluorescent aptasensors based on fluorescent dyes for the detection of antibiotics. (**A**) Scheme of a labeled fluorescent aptasensor for KAN detection based on structure switching. Adapted with permission from [17]. Copyright 2019 Frontiers. (**B**) Scheme of a labeled fluorescent aptasensor for CAP detection based on structure switching. Adapted with permission from [16]. Copyright 2020 Elsevier. (**C**) Schematic illustration of a labeled fluorescent aptasensor for CAP detection based on THMS. Adapted with permission from [25]. Copyright 2020 Elsevier. (**D**) Scheme of a label-free fluorescent aptasensor for KAN detection. Adapted with permission from [34]. Copyright 2020 Elsevier. (**E**) Schematic illustration of a label-free fluorescent aptasensor for TOB detection based on 3 WJ. Adapted with permission from [36]. Copyright 2021 Elsevier. (**F**) Schematic drawing for TET detection based on THMS and G-quadruplex. Adapted with permission from [26]. Copyright 2017 Elsevier.

The most frequently used labeled fluorescent aptasensors consist of two strands: the aptamer and the complementary strand, which are labeled with a fluorophore or quencher. In the absence of a target, the fluorophore and the quencher are in close proximity, and the FRET mechanism leads to fluorescence quenching. In the presence of a target, the aptamer specifically binds to the target owing to its high affinity. Then the complementary strands dissociate, resulting in fluorescence enhancement. Based on the above strategy, Ma and her co-workers designed a fluorescent aptasensor based on structure switching for the detection of kanamycin (KAN) (Figure 3A) [17]. The method was able to achieve highly sensitive and selective detection of KAN in a short time and was successfully applied to detection in actual samples. Benaissa et al. conducted research on truncated aptamer detection of ofloxacin (OFL). The optimal binding site of the 72-mer truncated aptamer to OFL was determined by computer simulation and molecular docking. Based on this, a rapid aptamer detection method based on FRET was developed. In the selectivity assay, OFL could be effectively distinguished from other quinolones and other antibiotics with similar structures [26].

Subsequently, Ma et al. put forward another fluorescence assay based on FRET for the rapid detection of chloramphenicol (CAP) (Figure 3B) [16]. This assay is composed of three ssDNA strands: one unlabeled aptamer and two complementary strands that are labeled with the fluorophore and quencher, respectively. Two complementary strands hybridize with the aptamer in the absence of CAP, forming a three-stranded structural switch. The fluorophore and quencher are in close proximity to each other, resulting in fluorescence quenching due to FRET. When CAP is present, it binds to the aptamer, thereby disrupting the aptamer-based structure switch. Simultaneously, the FRET efficiency between the fluorophore and the quencher is reduced, causing the recovery of fluorescence intensity. Whether it is a double- or triple-stranded DNA structural switch, it is crucial to optimize the complementary length and complementary position of the aptamer and cDNA. This will affect the sensing performance by influencing the background signal. Therefore, detailed optimization is required.

The triple helix molecular switch (THMS) exhibits distinct advantages over the double helix molecular switch, including high stability and preservation of the affinity and specificity of the aptamer. Babaei and colleagues devised a simple and quick method for identifying oxytetracycline (OTC) based on the THMS method (Figure 3C) [25]. In the absence of OTC, the aptamer and signal transduction probe (STP) form a THMS structure. Since the quencher is at a considerable distance from the fluorophore, the fluorescence intensity is high. The THMS structure disintegrates in the presence of OTC, and the STP forms a hairpin configuration, bringing the quencher and fluorophore closer together. As a result, FRET effectively quenches the fluorescence. The sequence lengths of the two arms at each end of the aptamer are essential to consider when designing the THMS in order to maintain the specificity of the aptamer to the target.

### 2.2. Label-Free Type

Despite the extensive application of fluorescently labeled aptasensors, the covalent labeling of aptamers with fluorophores and quenchers is time consuming and costly. Moreover, the label can reduce the aptamer’s affinity for its target, decreasing the sensitivity of the aptasensor. A large number of fluorescent aptasensors based on the label-free technique have been developed to solve these limitations. Label-free reporters are generally cationic conjugated polymers or free dyes that intercalate into various aptamer structures and generate fluorescence signals in the presence or absence of the target.

It is widely recognized that berberine is an isoquinoline alkaloid isolated from rhizoma copies with extensive biochemical and pharmacological activities [38]. X-ray diffraction experiments verified that berberine binds to DNA utilizing an intercalation interaction. At present, berberine is widely used as a DNA-binding agent for the construction of fluorescent aptasensors. Zhou and his co-workers developed a highly sensitive fluorescent aptasensor for detecting KAN (Figure 3D) [34]. Berberine was utilized as a fluorescence reporter, while the KAN-aptamer was used as the detection unit in the fluorescent aptasensor. Berberine fluorescence is increased when it combines with the aptamer. The randomly coiled structure of the KAN-aptamer turns into a hairpin configuration once KAN is detected by the aptasensor, causing berberine to dissociate from the KAN-aptamer. This leads to fluorescence quenching.

PicoGreen (PG) reagent is an asymmetric cyanine dye that exhibits no fluorescence when it is in isolation [39]. Upon binding to double-stranded DNA (dsDNA), its fluorescence intensity increases by almost 1000-fold. To detect tobramycin (TOB), Khajavian et al. developed a fluorescence assay based on the creation of a three-way junction (3WJ) structure using a highly sensitive and straightforward technique (Figure 3E) [36]. The number of double-stranded sequences changes in the presence of TOB, resulting in a difference in PG fluorescence signal intensity.

Unlike PG, thioflavin (ThT) in its free state exhibits a significant increase in fluorescence intensity when bound to the G-quadruplex [40,41]. To detect tetracycline (TET), Chen and his colleagues developed a fluorescent aptasensor based on a G-quadruplex and THMS (Figure 3F) [30]. The structural switch consists of an 8-mer aptamer sequence and two flanking arm segments. The detection limit is 970.0 pmol/L, which is lower than the detection limits for TET using CE, electrochemical, and HPLC techniques. However, the selectivity of this aptasensor is low, which might be attributed to alteration of the TET-aptamer. The weaknesses of the above strategy include time-consuming operation processes and extended analysis durations.

Overall, fluorescent dye-based fluorescent aptasensors have the advantages of simplicity of operation, feasibility of methodology, etc., but there are disadvantages such as poor stability and high price.

## 3. Nanomaterials in Fluorescent Aptasensors for the Detection of Antibiotics

Aptasensors utilizing nanomaterials generally exhibit better stability in comparison to fluorescent dyes. Nanomaterials are materials with at least one dimension size ranging from 1 nm to 100 nm [42,43]. Owing to their large specific surface area, good biocompatibility, and excellent optical properties, they have been widely used in the construction of fluorescent aptasensors. Moreover, nanomaterials demonstrate many important advantages in improving the recognition rate of specific targets, reducing the analysis time, and enhancing signal readout. A comprehensive overview of the different fluorescent aptasensors based on nanomaterials for the detection of antibiotics in food is presented in Table 3.

### 3.1. Upconversion Nanoparticles

**Figure 4:**
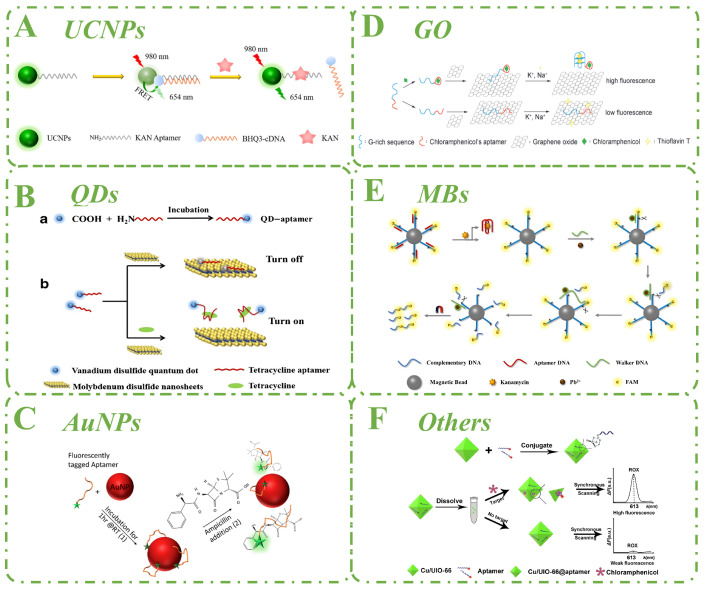
Fluorescent aptasensors based on nanomaterials for the detection of antibiotics. (**A**) Scheme of a fluorescent aptasensor based on UCNPs and BHQ3. Adapted with permission from [22]. Copyright 2021 Elsevier. (**B**) Scheme of a fluorescent aptasensor based on QDs and MoS_2_ nanosheets. Adapted with permission from [54]. Copyright 2019 Elsevier. Schematic representation of the preparation of aptamer labeled VS2 QD (**a**) for aptamer-based fluorometric tetracycline assay (**b**). (**C**) Scheme of a fluorescent aptasensor based on AuNPs and FAM. Adapted with permission from [60]. Copyright 2020 MDPI. (**D**) Scheme of a fluorescent aptasensor based on GO and ThT. Adapted with permission from [61]. Copyright 2020 Elsevier. (**E**) Scheme of a fluorescent aptasensor based on MBs and FAM. Adapted with permission from [62]. Copyright 2020 Elsevier. (**F**) Scheme of a fluorescent aptasensor based on MOFs and ROX. Adapted with permission from [65]. Copyright 2020 SPRINGER.

Lanthanide-doped up-conversion nanoparticles (UCNPs) possess the ability to convert lower-energy near-infrared excitation into higher-energy visible light emission. Moreover, UCNPs can effectively avoid autofluorescence interference from the sample matrix during optical excitation at the near-infrared wavelength of 980 nm and achieve high-sensitivity analysis. UCNPs also offer several additional advantages, including chemical stability, low toxicity, easy wavelength tuning, deep penetration in living tissues, and photobleaching resistance, making them more competitive than other luminescent materials for analytical purposes. Thus, they have been used to develop sensors for food assays, medical diagnostics, environmental hazard detection, and biological analysis. Chen et al. developed a simple FRET-based aptasensor for the detection of KAN in food samples that is both selective and sensitive (Figure 4A) [22]. The UCNP-aptamer hybridizes with BHQ3-cDNA in the absence of KAN, and the fluorescence is quenched due to the FRET effect between BHQ3 and UCNPs. KAN binds to the aptamer when present, and the double chain splits. As a result, the UCNPs and BHQ3 are separated, and the fluorescence intensity is recovered. Even in the presence of structurally identical and different antibiotics, the constructed aptasensor exhibited good selectivity for KAN. Furthermore, the designed aptasensor could detect KAN in real samples. Chen et al. continued the research on UCNPs [48]. Using the broadband absorption properties of MnO_2_ nanosheets and luminescence resonance energy transfer (LRET) with UCNPs, a method was developed for the rapid and sensitive detection of TET in food samples. The detection of other targets can be achieved by changing aptamers, and the simultaneous detection of multiple targets can be achieved using UCNPs.

### 3.2. Quantum Dots

Quantum dots (QDs), a widely favored class of fluorescent nanomaterials, have been utilized to make a range of optical probes that have several advantages over fluorescent dyes, including high quantum yield, broad excitation spectra, size-dependent characteristics, and good anti-bleaching capabilities. Zhang et al. developed an aptasensor using FRET between nitrogen-doped graphene quantum dots (N-GQDs) and hydroxyl cobalt oxide (CoOOH) nanosheets to achieve the detection of TET in the linear range of 1 to 100 ng mL^−1^ with a detection limit of 0.95 ng mL^−1^ [52]. Ma and coworkers designed a fluorescent aptasensor for the detection of TET using molybdenum (IV) disulfide (MoS_2_) nanosheets as an effective quencher and VS_2_ QDs as excellent fluorescent probes (Figure 4B) [54]. This aptasensor enables selective and sensitive TET bioassays over a linear range of 1 to 250 ng mL^−1^ with a limit of detection is 0.06 ng mL^−1^.

### 3.3. Gold Nanoparticles

Gold nanoparticles (AuNPs) exhibit remarkable properties, including high surface-to-volume ratios, tolerance to various sulfhydryls, size-dependent optical and electronic properties, and they are frequently utilized in the fabrication of fluorescent aptasensors with concomitant fluorescence bursting ability, catalytic capacity, and chemical stability. Song et al. developed a simple and rapid fluorescent assay for detecting ampicillin (AMP) (Figure 4C) [60]. In the absence of AMP, the FAM-labeled aptamer is non-specifically bound to AuNPs, which results in weakening of the fluorescence intensity. As the concentration of AMP in the solution increases, the fluorescence intensity gradually increases as AMP binds to the aptamer and moves away from the AuNP surface.

### 3.4. Graphene Oxide

Graphene oxide (GO), a novel two-dimensional carbon nanomaterial with unique physicochemical properties, possesses a superior application prospect in the fabrication of colorimetric, electrochemical, and fluorescent aptasensors due to its excellent water solubility, biocompatibility, and fluorescent quenching ability. It can interact with numerous organic, inorganic, and biological compounds via noncovalent, covalent, or electrostatic interactions after being added to the sensing platform, resulting in better selectivity and sensitivity of aptasensors. Based on GO and G-rich aptamer sequences, Lu et al. developed a label-free and rapid fluorescent aptasensor for the detection of CAP (Figure 4D) [61]. The aptamer is adsorbed on the GO surface in the absence of CAP, inhibiting the formation of the G-quadruplex structure and resulting in low fluorescence intensity in the system. The aptamer moves away from GO in the presence of CAP and forms a G-quadruplex structure with the help of sodium and potassium ions. When the fluorescent dye ThT attaches to the G-quadruplex, the system’s fluorescence intensity is dramatically increased.

### 3.5. Magnetic Beads

Magnetic beads (MBs) are recognized for enhancing detection sensitivity and selectivity due to their high surface-to-volume ratio, diverse size-dependent properties, rapid kinetics, and ease of use. Conjugating biomolecules to the surface of MBs is also straightforward and flexible. More importantly, magnetic separation effectively reduces interference from the complex matrix, resulting in excellent sensitivity and accuracy. For the detection of KAN, Yang et al. developed a sensitive fluorescent technique based on a DNA walker (Figure 4E) [62]. Initially, the aptamer is hybridized partially with the cDNA modified on MBs. When KAN is present, it binds to the aptamer, causing the cDNA to hybridize with the walker DNA. The cleavage of recognition sites by DNAzyme then drives the autonomous mobility of DNA walkers on the surface of MBs. The signal probe is isolated, and an amplified fluorescent signal is generated by the accumulation of the signal probe.

### 3.6. Other Nanomaterials

In addition to the aforementioned nanomaterials, many other remarkable nanomaterials are frequently utilized in the fabrication of fluorescent aptasensors, such as metal–organic frameworks (MOFs), silver nanoclusters (AgNCs), carbon nanotubes (CNTs), and so on. Lu et al. successfully developed the bimetallic organic framework nanomaterial Cu/UiO-66 and established a fluorescence-based technique for the quantitative detection of CAP (Figure 4F) [65]. All single-stranded aptamers are adsorbed on the surface of Cu/UiO-66 in the absence of CAP, bringing ROX into close proximity with Cu/UiO-66. The ROX fluorescence is then quenched by Cu/UiO-66. In the presence of CAP, however, CAP interacts with the aptamer to produce a unique spatial structure in which the ROX is displaced from the Cu/UiO-66 surface due to a change in the aptamer’s spatial structure, allowing the ROX fluorescence to be recovered. Low fluorescence background, great sensitivity, and good reproducibility are all advantages of this approach.

Overall, nanomaterial-based aptasensors can effectively reduce costs and improve detection performance, but problems such as the complexity of the preparation procedure and the consistency of material batches need to be resolved.

## 4. Aptamer Detection Technology Performance Enhancement Strategies

Although many antibiotics can be adequately quantified by the fluorescent sensors described above, some antibiotics require high detection performance that cannot be achieved by existing conventional detection methods. Therefore, by combining other techniques such as nucleic acid tailoring, signal amplification, and artificial intelligence (AI) techniques, the sensitivity can be greatly improved to meet the needs of trace detection.

### 4.1. Nucleic Acid Tailoring Strategy

**Figure 5:**
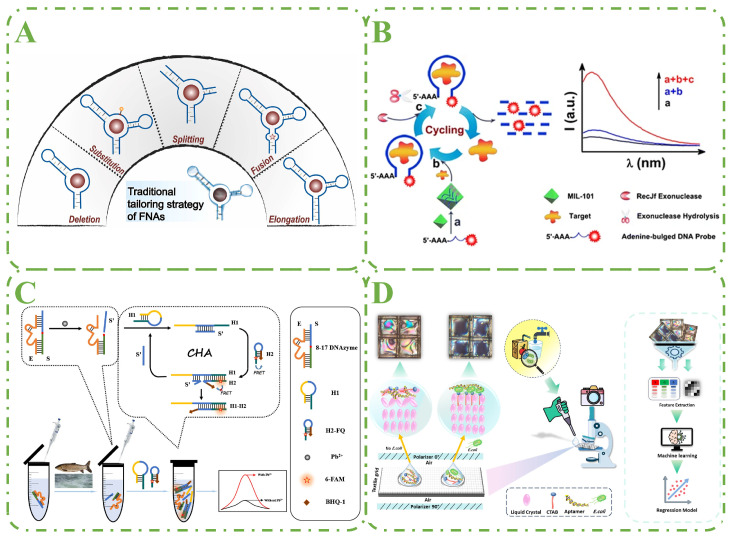
Aptamer detection technology performance enhancement strategies. (**A**) Nucleic acid tailoring strategy. Adapted with permission from [66]. Copyright 2024 Elsevier. (**B**) Schematic diagram of the aptamer-involved fluorescence amplification strategy facilitated by directional enzymatic hydrolysis. Adapted with permission from [67]. Copyright 2017 SPRINGER. (**C**) Schematic diagram of the structure switching aptamer triggering enzyme-free amplification strategy for TOB detection based on HCR and fluorescence synergism. Adapted with permission from [68]. Copyright 2022 Elsevier. (**D**) AI-assisted aptamer performance enhancement. Adapted with permission from [69]. Copyright 2022 Springer.

The structurally diverse characteristics of nucleic acids give them excellent compatibility with different targets. Therefore, the inherent designability of aptamers can be exploited to modulate their assay performance through tailoring [66]. A commonly used strategy is to artificially manipulate their sequences and structures, with the main objective of improving the binding affinity and recognition performance of the aptamers. In general, aptamer tailoring strategies can be classified into five categories, including deletion, substitution, splitting, fusion, and extension (Figure 5A). It facilitates enhanced cost-effectiveness, binding affinity, and generation of new functional sequences.

### 4.2. Enzyme-Assisted Signal Amplification

Aptamers can be manipulated and amplified by a wide range of enzymes, including ligases, polymerases, exonucleases, and endonucleases. Enzyme-assisted signal amplification approaches use enzyme-specific activities, carefully designed primers, and templates to regularly reproduce the target nucleic acid under precise physical and chemical conditions to achieve signal amplification. Common enzyme-assisted signal amplification methods include rolling circle amplification (RCA), strand displacement reaction (SDA), and polymerase chain reaction (PCR).

He et al. constructed a fluorescent assay to quantify OTC (Figure 5B). The enzyme-induced target cycle signal amplification test was used in conjunction with a metal–organic framework platform. The fluorescence of DNA probes is first quenched by MIL-101. After being introduced to MIL-101, the DNA probe binds to a specific target and then desorbs. The fluorescence is gradually restored. RecJf hydrolysis from the 5′ to the 3′ terminal uses the DNA probes as a substrate right away. On MIL-101, the target is freed and begins to bond with additional DNA probes. As a result, the following wave of cleavage begins, resulting in a considerable amplification of the fluorescence signal [67]. Zhou and his colleagues developed a highly sensitive fluorescence technique for detecting KAN in real samples [70]. To begin, KAN binds the aptamer hybridized with the hairpin and commences the primer exchange reaction process under isothermal circumstances, using Bst-DNA polymerase to produce Mg^2+^-dependent DNAzyme autonomously. This type of synthesis can be performed several times, yielding a vast number of DNAzymes that cyclically cleave the rA sites in the signal hairpin substrate with the help of Mg^2+^ cofactors, releasing a large number of free G-quadruplex fragments. ThT binds to these G-quadruplex fragments, producing considerably increased fluorescence for KAN detection.

### 4.3. Enzyme-Free Signal Amplification

Because the temperature and other reaction conditions have a big impact on enzyme activity, enzyme-free signal amplification has an advantage over enzyme-assisted signal amplification methods. To date, the most well-established enzyme-free signal amplification techniques include catalytic hairpin assembly (CHA) and hybridization chain reaction (HCR).

Based on CHA and G-quadruplex displacement, Zhou and colleagues suggested a fluorescent technique for detecting TET [71]. First, HP1 and HP2 hairpin probes were created, with the G-rich HP1 loop area forming a stable G-quadruplex structure that can bind to the fluorescent dye NMM and emit intense fluorescence. When TET is present, it can bind to the aptamer at the sticky end of HP1, causing CHA to be triggered. This approach achieves quantitative detection of TET due to the drop in fluorescence intensity produced by the change in the G-quadruplex structure.

By injecting initiator single-stranded DNA and generating DNA nanostructures with lengthy repeat units, HCR is an enzyme-free isothermal amplification approach based on toehold-mediated strand displacement that can amplify the output signal hundreds of times. Based on HCR and fluorescence synergism, Wang et al. fabricated a novel aptasensor for the detection of TOB (Figure 5C). TOB separates cDNA-FAM from the magnetic beads by competing for binding to the aptamer. After magnetic separation, cDNA-FAM triggers HCR. Then the fluorescence of the system is significantly enhanced by the synergistic effect of SGI and FAM. In addition, the background fluorescence can be significantly reduced due to the incorporation of GO. Signal amplification aptasensors can be applied to the detection of other targets by replacing the aptamer sequence [68].

### 4.4. AI-Assisted Aptamer Performance Enhancement

Aptamers are often screened by exponentially enriched ligand systematic evolution techniques (SELEX). However, due to the limitations of sequencing capacity and experimental conditions, traditional SELEX methods can only evaluate a limited number of sequences and cannot adequately explore the theoretical sequence space of huge nucleotides, resulting in the omission of many potentially efficient aptamers. Although aptamer screening methods have been continuously improved, and improved SELEX techniques such as CE-SELEX, capture-SELEX, and GO-SELEX have been derived, these methods usually require repeated screening and amplification, with a long experimental period and high cost. AI can assist in generating and optimizing high-affinity aptamers, which improves the efficiency of screening and reduces the production cost to a certain extent [72]. The rapid development of AI-based methods and the availability of big data have paved the way for the application of AI-based methods (e.g., neural networks and deep learning) in aptamer screening and performance enhancement (Figure 5D) [69].

## 5. Conclusions and Future Perspectives

This review provides a comprehensive summary of the published work on fluorescent aptasensors for the detection of antibiotics in recent years. Various fluorescent aptasensors based on fluorescent dyes and nanomaterials are described in detail, as well as several techniques for aptamer detection performance enhancement to improve analytical sensitivity.

However, there are still some challenges in the practical application of fluorescent aptasensors in the detection of antibiotics. (1) Although aptamers can theoretically detect any antibiotic, the limited number of aptamers identified to date may limit their application. (2) Fluorescent aptasensors often have problems such as excessive background fluorescence or self-fluorescence, which seriously affect their detection performance. (3) The current research rarely works for real samples or reaches the level of commercialization. This is because real samples are extremely complex and detection may be affected by unexpected events.

To address these challenges, we offer the following suggestions. (1) There is a need to screen for a wider variety of aptamers with stronger affinity in combination with artificial intelligence and other technologies. (2) As the introduction of functional nanomaterials can enhance the sensing performance, there is a need to develop new fluorescent dyes or nanomaterials with stronger stability, signal transduction ability, and sensing ability. (3) Development of enzyme-free or nanomaterial-based signal amplification strategies to improve sensor sensitivity and save costs. (4) Efficient sensing devices with better biocompatibility are necessary for the successful detection of food targets in complex sample matrices in practical applications. (5) We look forward to transferring more work to practical sensing devices that take sample pretreatment into account to facilitate the commercialization and practical application of fluorescent sensors.

We conclude the article by appreciating the published work on fluorescent aptasensors for antibiotic detection and look forward to the future emergence of more sophisticated aptasensors. We hope that this review provides readers with an overview of current developments in the field and inspires them to come up with new ideas for future research.

## Figures and Tables

**Table 2:** Overview of fluorescent aptasensors based on fluorescent dyes for the detection of antibiotics.

	Analyte	Materials	Linear Range	LOD	Sample	Reference
Labeled	TOB	SGI	0.1–6 μM	0.063 μM	Milk	[12]
	CAP	FAM/BHQ1	3–309 nM	2.16 nM	Milk	[16]
	KAN	FAM/Dabcyl	100–600 nM	13.52 nM	Milk	[17]
	KAN	ThT	0.6–20 nM	0.33 nM	Milk	[18]
	TET	TO	0.11–225 μM	0.07 μM	Milk	[19]
	OTC	FAM/BHQ1	0–250 nM	1.67 nM	Milk	[23]
	KAN	Cy5/Cy3	0.05–5 nM	0.18 nM	Milk	[24]
	CAP	FAM/BHQ1	5–200 nM	1.2 nM	Honey	[25]
	OFL	FAM/TAMRA	0.2–20 μM	0.12 μM	Milk	[26]
	TET	Pyrenes	5.0–100 nM	1.6 nM	Milk	[27]
Label-free	KAN	NMM	0.5–100 nM	0.5 nM	Milk	[28]
	KAN	ThT	1 nM–300 uM	300 pM	Milk	[29]
	TET	ThT	0.2–20.0 nM	970.0 pM	Serum	[30]
	KAN	ThT	0.7–10 nM	0.37 nM	Milk	[31]
	TET	SGI	11–56 μM	0.23 μM	Milk	[32]
	OFL	SGI	1.1–200 nM	0.34 nM	Water	[33]
	KAN	Berberine	5.0–71.0 nM	2.3 nM	Milk	[34]
	TET	ThT	0.01–1.0 μM	0.001 μM	Honey	[35]
	TOB	PG	80 nM–2 μM	21.86 nM	Serum	[36]
	KAN	ThT	50–2000 nM	1.05 nM	Pork	[37]

**Table 3:** Overview of fluorescent aptasensors based on nanomaterials for the detection of antibiotics.

Analyte	Materials	Linear Range	LOD	Sample	Reference
TET	UCNPs/MNPs	0.02–225 nM	0.014 nM	Pork	[20]
SLF	UCNPs/MNPs	3–29 nM	0.35 nM	Perch	[21]
SMZ	UCNPs/Au@Ag/AuNPs	0.36–359 nM	0.07 nM	Milk	[44]
ENR	UCNPs/GO	2–173 nM	1.3 nM	Milk	[45]
KAN	UCNPs/BHQ3	0.005–50 uM	18.9 nM	Milk	[22]
AMP	UCNPs/PtNPs	1–247 nM	0.79 nM	Pork	[46]
TET	UCNPs/MNPs	1–2250 nM	0.38 nM	Pork	[47]
TET	UCNPs/MnO_2_ nanosheets	0.02–225 nM	0.019 nM	Milk	[48]
CAP	QDs/AuNPs	0.015–309 nM	9 pM	Milk	[49]
KAN	QDs/BHQ1	0.02–185 nM	12 pM	Milk	[50]
SDM	QDs/PDDA	80–966 nM	7.21 nM	Fish	[51]
TET	QDs/CoOOH nanoflakes	2–225 nM	2.13 nM	Milk	[52]
KAN	QDs/AuNPs	0.01–500 nM	5.7 pM	Milk	[53]
TET	QDs/MoS_2_ nanosheets	2–562 nM	0.13 nM	Milk	[54]
OFL	RB/AuNPs	20–300 nM	1.66 nM	Milk	[55]
AMP	PTCDI/AuNPs	100–1000 pM	29.2 pM	Serum	[56]
SDM	SGI/AuNPs	6–966 nM	10.98 nM	Fish	[57]
KAN	FAM/AuNPs	0.1 pM–0.1 uM	0.1 pM	Water	[58]
SMZ	RB/AuNPs	4–143 nM	2.94 nM	Water	[59]
AMP	FAM/AuNPs	100 nM–100 μM	20.6 nM	Urine	[60]
CAP	FAM/GO	6–618 nM	0.9 nM	urine	[61]
CAP	ThT/GO	2–20 nM	1.45 nM	Milk	[62]
CAP	SG/MOFs	0.003–30 nM	0.9 pM	Milk	[63]
KAN	RB/MSNs	24.75–137.15 nM	7.5 nM	Serum	[64]
CAP	ROX/MOFs	0.2–10 nM	0.09 nM	Fish	[65]

## Abbreviations

The following abbreviations are used in this manuscript:

ENR	Enrofloxacin
MSNs	Mesoporous silica nanoparticles
NMM	N-methylmesoporphyrin IX
OFL	Ofloxacin
PtNPs	Platinum nanoparticles
RB	Rhodamine B
SG	SYBR gold
SGI	SYBR Green I
SLF	Sulfadimethoxine
SMZ	Sulfamethazine
TO	Thiazole orange
